# Hsa_circ_0000515 sequesters microRNA‐296‐5p and elevates RNF44 expression to encourage the NSCLC progression

**DOI:** 10.1002/ccs3.70005

**Published:** 2025-02-24

**Authors:** Lixin Sun, Bei Lu, Chongyuan Li, Guangquan Xu

**Affiliations:** ^1^ Department of Thoracic Surgery The Fourth Affiliated Hospital of Harbin Medical University Harbin China; ^2^ Department of Thoracic Surgery The Second Affiliated Hospital of Harbin Medical University Harbin China

**Keywords:** hsa_circ_515, microRNA‐296‐5p, non‐small cell lung cancer, RNF44

## Abstract

Circular RNAs (circRNAs) are RNA molecules frequently involved in tumorigenesis. This research study focuses on the relevance of hsa_circ_515 (circ_515) to non‐small cell lung cancer (NSCLC) progression and the downstream targets involved. Differentially expressed circRNAs in NSCLC were screened using a GSE158695 dataset. Circ_515 was overexpressed and indicated poor outcomes in patients with NSCLC. Knockdown of circ_515 repressed proliferation and invasiveness, while potentiated cell cycle arrest and apoptosis of NSCLC cells, and upregulation of circ_515 led to converse trends. The candidate downstream transcripts of circ_515 were explored using integrated bioinformatic analyses. Ectopic expression of miR‐296‐5p reduced the malignance of NSCLC cells. Circ_515 sequestered miR‐296‐5p and blocked its suppressive role in RING finger protein 44 (RNF44) expression. Downregulation of RNF44 counteracted the oncogenic effects of circ_515. In vivo, the anti‐tumor effects of circ_515 knockdown were reversed by miR‐296‐5p, while the tumor‐promoting effects of circ_515 upregulation were abolished by RNF44 knockdown. All in all, our findings demonstrate that circ_515 sequesters miR‐296‐5p and elevates RNF44 expression to encourage the NSCLC progression. This study might provide new thoughts on NSCLC management.

## Graphical abstract

In this study, a novel circular RNA_515 (circ_515)/microRNA‐296‐5p (miR‐296‐5p)/RING finger protein 44 (RNF44) axis is identified to be closely linked to the pathogenesis of non‐small cell lung cancer (NSCLC). Circular RNA_515 may serve as a novel prognostic biomarker for NSCLC cells whose upregulation enhanced growth and invasiveness of cancer cells through sponging miR‐296‐5p and activating RNF44.

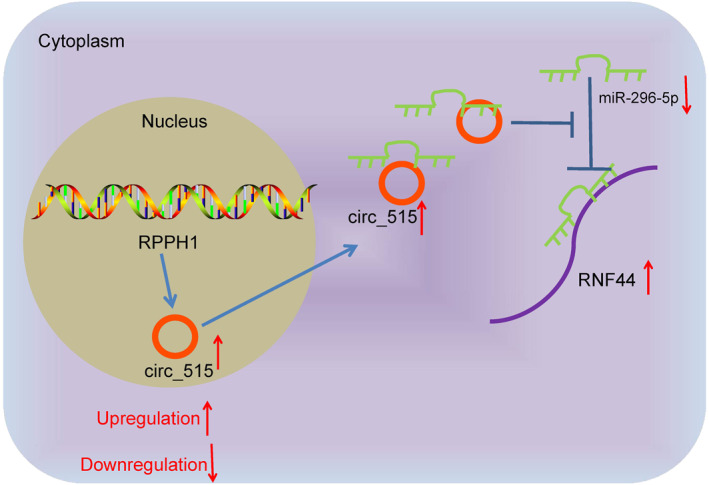

## 1 INTRODUCTION

With a projected 2.2 million new diagnoses and 1.8 million deceases in 2020, lung cancer (LC) represents the most frequent and life‐threatening cancer in the world.^1^ Non‐small cell LCs (NSCLCs) represent a large subgroup that accounts for about 85% of all LC cases, and they can further be allocated into several cancer types, including squamous cell cancers (LUSC), adenocarcinoma (LUAD), and large cell cancers.^2^ Smoking remains the predominant risk factor for both NSCLC and small cell lung cancer.^3^ Conventional treatments including surgery, radiotherapy, chemotherapy, and immunotherapy remain the most common therapeutic options for NSCLC patients.^4^ However, because of the absence of early signs, most patients are not diagnosed until advanced stages with local or distant metastasis, and the 5‐year survival of LC patients with advanced disease is dismal at approximately 5%.^5^ A better understanding of the disease development is required to develop a novel therapeutic regimen.

About 97% of all human genomes are transcribed to noncoding (nc)RNAs that play fundamental functions in tumorigenesis.^6^ Among them, circular RNAs (circRNAs) have been revealed as major mediators of gene expression and biological functions.^7^ CircRNAs are highly stable RNAs that are produced from pre‐mRNAs through back‐splicing.^8^ This special class of transcripts can also lead to microRNA (miRNA) inhibition and diminish its suppressive function on other transcripts, termed competitive endogenous RNAs.^9^, ^10^ They have been frequently reported as key biomarkers or regulators in NSCLC and are relevant to an array of events, including cell development, metastasis, and drug resistance.^11^ In this paper, according to an analysis of a GSE158695 dataset (circRNA expression data from 3 human NSCLC tissues and the corresponding noncancerous tissues), we observed that only circ_001678 has been reported in NSCLC among the six top circRNAs (top three regarding adjusted *p*‐value and top three regarding the LogFC value).^12^ As for the remaining five circRNAs, we noticed hsa_circ_515 (also known as hsa_circ_000585; hereafter designated as circ_515) because it is the only overlapping one in the two lists. Circ_515 is located at chr14:20,811,305–20811,534 and its host gene is ribonuclease P RNA component H1 (RPPH1) (http://www.circbase.org/cgi‐bin/singlerecord.cgi?id=hsa_circ_0000515). It is a rarely studied circRNA. Even though only one research reported its high expression in tumor tissues of cholangiocarcinoma,^13^ the role of circ_RPPH1 has been revealed in breast cancer and glioma.^14^, ^15^ Nevertheless, the exact function of circ_515 in NSCLC development has not been established. Our subsequent bioinformatic analyses revealed circ_515 possibly sponges miR‐296‐5p and restored the expression of ring finger protein 44 (RNF44). MiR‐296‐5p has been found to halt proliferation, cell cycle progression, and drug resistance in NSCLC by targeting different mRNA targets.^16^, ^17^ RNF44 is an E3 ligase associated with AMPK‐α1 degradation in BRAFi‐resistant melanoma cells.^18^ Taken together, we postulated that circ_515 possibly regulates RNF44 expression by sponging miR‐296‐5p in NSCLC. This study was therefore performed to analyze the interactions between circ_515, miR‐296‐5p, and RNF44 and their impact on the malignant properties of NSCLC cells through a series of in vitro and in vivo assays.

## 2 MATERIALS AND METHODS

### 2.1 Ethics

The study was approved by the Ethical Committee of the Fourth Affiliated Hospital of Harbin Medical University. An informed consent form was obtained from each respondent. Animal procedures were approved by the Animal Committee of the Fourth Affiliated Hospital of Harbin Medical University and performed strictly adhering to the Guide for the Care and Use of Laboratory Animals (NIH, Bethesda).

### 2.2 Microarray analysis

A GSE158695 dataset (https://www.ncbi.nlm.nih.gov/geo/query/acc.cgi?acc=GSE158695) based on the GPL19978 Agilent‐069978 Arraystar Human CircRNA microarray V1 platform was downloaded. The dataset comprises data on circRNA expression from three NSCLC tissues and three normal lung tissues. An R Limma Package (https://git.bioconductor.org/packages/limma) was used for data processing. Differentially expressed (DE) genes between NSCLC tissues and normal lung tissues were screened using |log2 (Fold Change) | > 2 and *p* < 0.05 as the thresholds.

### 2.3 Clinical sample collection

From February 2014 to January 2015, 50 NSCLC patients admitted to the Fourth Affiliated Hospital of Harbin Medical University were enrolled. The NSCLC and the para‐tumorous tissues were collected by surgical resection. All participants were first diagnosed with NSCLC. They did not have a history of chemotherapy or radiotherapy or have any other malignancies. The tissue samples were immediately frozen at −80°C. These patients were followed up for 5 years at 6‐month intervals to monitor the prognosis of patients.

### 2.4 Cell culture

Human NSCLC cells A549 (CCL‐185), NCI‐H1299 (CRL‐5803), SK‐MES‐1 (HTB‐58), Calu‐3 (HTB‐55), and a bronchial epithelial cell line HBEC3‐KT (CRL‐4051) were obtained from ATCC. All cells were grown in RPMI‐1640 medium (Gibco Company) plus 10% fetal bovine serum and 1% P/S at 37°C with 5% CO_2_.

### 2.5 Reverse transcription‐quantitative polymerase chain reaction (RT‐qPCR)

Total RNA from tissues or cells was extracted from the TRIzol reagent. The NanoDrop 3300 (Thermo Fisher Scientific) was used for RNA purity and concentration assessment. The RNA was reverse‐transcribed into cDNA using the reverse transcriptase SuperScript II (Invitrogen), and AceQ qPCR SYBR Green Master Mix (Vazyme Biotech Co. Ltd.) was used for qPCR on an ABI 7500 qPCR system (Applied Biosystems Inc.). GAPDH and U6 were used as the endogenous loadings. The primer sequences are listed in Table 1.

**TABLE 1:** Primer sequences for RT‐qPCR.

Primers	Sequence (5′‐3′)
circ_515	F: GGTCAGACTGGGCAGGAG
	R: CTGTTAGGGCCGCCTCTG
miR‐296‐5p	F: CCCCCCCTCAATCCTG
	R: GAACATGTCTGCGTATCTC
RNF44	F: GATGTTCAGTGGGCAGCATTACC
	R: GCAGGATGTAGTGGTCACTGGA
GAPDH	F: GGAGCGAGATCCCTCCAAAAT
	R: GGCTGTTGTCATACTTCTCATGG
U6	F: CTCGCTTCGGCAGCACA
	R: AACGCTTCACGAATTTGCGT

Abbreviations: circRNAs, circular RNAs; GAPDH, glyceraldehyde‐3‐phosphate dehydrogenase; miR, microRNA; RT‐qPCR, reverse transcription‐quantitative polymerase chain reaction; RNF44, ring finger protein 44.

### 2.6 Detection of circ_515 stability by RNase R treatment

RNA was treated with RNase R (Applied Biological Materials [ABM] Inc.). The RNA samples extracted from A549 and SK‐MES‐1 cells were equally allocated to two portions. For one portion (treated with RNase R), 2 μg total RNA was mixed with 2 μL 10 × RNase R reaction buffer and 2 μL RNase R (20 U/μL). The RNase R was replaced by an equal volume of diethyl pyrocarbonate‐treated water for the other portion. After a 0.5‐h water bath at 37°C, the expression of circ_515 and RPPH1 was assessed by RT‐qPCR.

### 2.7 Western blot (WB) analyses

Cells were lysed in radio immunoprecipitation assay lysis buffer (Beyotime) to extract total protein. After concentration determination, the protein samples were separated by 12% SDS‐PAGE and loaded onto polyvinylidene fluoride membranes (Millipore Corp.). After being sealed with 5% nonfat milk, the membranes were co‐cultured with primary antibodies overnight at 4°C and with secondary antibodies at 20°C for 2 h. The ECL Plus western blotting substrate (Thermo Fisher Scientific) was applied for visualization, and the quantification was conducted using Image J software. First, the western blot images were converted into grayscale images, and after removing the background, the images were converted into bright bands with set quantitative parameters and units. The next step was to circle the area of selected bands to measure the grayscale value. The relative expression of the target protein was defined as the rate of the gray value of the target protein to that of GAPDH. Antibodies used are listed in Table 2.

**TABLE 2:** The antibody information used in WB.

Antibodies	Catalog number and manufacture	Dilution
E‐cadherin	ab40772, Abcam	1:10,000
Vimentin	ab92547, Abcam	1:1000
Snail	ab216347, Abcam	1:1000
GAPDH	#5174, Cell Signaling Technologies	1:1000
Goat anti‐rabbit IgG H&L (HRP)	ab97051, Abcam	1:20,000

### 2.8 Nuclear‐cytoplasmic RNA separation

Subcellular localization of circ_515 was examined. The nuclear and cytoplasmic RNA was isolated using a PARIS kit (Invitrogen). The expression of circ_515 in nuclear and cytoplasmic RNA was examined by RT‐qPCR using U6 and GAPDH as the controls, respectively.

### 2.9 RNA pull‐down assay

The biotin‐conjugated probe of circ_515 and the control probe (Bio‐NC) were synthesized by RiboBio. The lysates of 1 × 10^7^ NSCLC cells were incubated with bio‐circ‐ or bio‐NC‐conjugated streptavidin magnetic beads at 4°C overnight. After that, the combined RNA was washed in a washing buffer and purified for RT‐qPCR.

### 2.10 In vivo assays

Male BALB/c nude mice (4–6 weeks, Vital River) were allocated into 8 groups: sh‐NC, sh‐circ_515, oe‐NC, oe‐circ_515, sh‐circ_515 + NC inhibitor, sh‐circ_515 + miR‐296‐5p inhibitor, oe‐circ_515 + sh‐NC, and oe‐circ_515 + sh‐RNF44 (*n* = 5 in each group). The mixture of SK‐MES‐1 and A549 cells and 30% Matrigel (1 × 10^6^ cells in 100 μL) was subcutaneously injected into mice. The volume (*V*) of tumors was monitored every 5 days as follows: *V* = *a* × b^2^ × 0.5. On the 30th day, the mice were sacrificed using 150 mg/kg pentobarbital sodium (*i.p*.) to harvest the tumors for weighing and further analysis.

### 2.11 Statistical analyses

Prism 8.02 (GraphPad) was applied. Differences were analyzed by the *t‐test* for two groups or one‐ or two‐way ANOVA with Sidak’s or Tukey’s adjustment for multiple groups. All data were presented as mean ± SD. The log‐rank test was applied for survival analysis. The clinical characteristics were analyzed by Fisher’s exact test. A significant difference was set at *p < 0.05.

Additional methods, including cell transfection, CCK8 assay, colony formation assay, flow cytometry, wound healing assay, transwell assay, immunohistochemical assay (IHC), and dual‐luciferase activity assays were detailed in Supporting Information S1.

## 3 RESULTS

### 3.1 Circ_515 is enhanced in NSCLC patients and predicts an unfavorable prognosis

**FIGURE 1:**
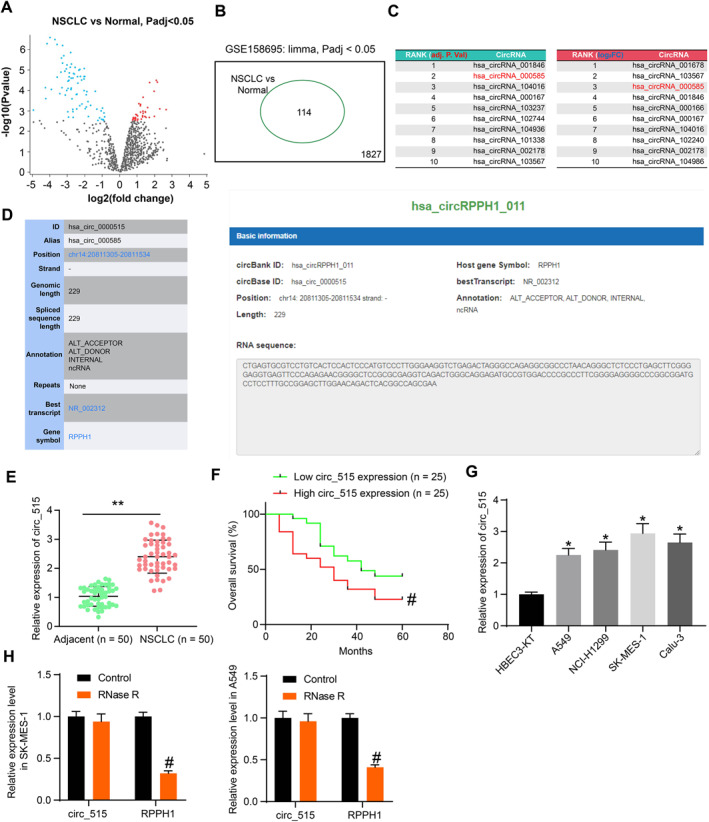
Circ_515 is highly expressed in NSCLC tissues and cells. (A and B), volcano plots (A) and the number (B) of the differentially expressed circRNAs in the NSCLC dataset GSE158695; (C), ranks of differentially expressed elevated circRNAs in the GSE158695 dataset by both adj. *p*. value and LogFC; (D), information of hsa_circ_000585 in the circBase system; (E), circ_515 expression in the NSCLC and the adjacent tissues quantified by RT‐qPCR; (F), the correlation between circ_515 and the survival of patients by log‐rank test; (G), circ_515 expression in NSCLC cell lines (A549, NCI‐H1299, SK‐MES‐1 and Calu‐3) and normal HBEC3‐KT cells examined by RT‐qPCR; (H), circ_515 stability in NSCLC cells examined by RNase R treatment. Data were collected from three independent experiments and expressed as mean ± SD. Differences were analyzed by paired *t‐test* (E); one‐way ANOVA and Dunnet's multiple comparison tests (G) or two‐way ANOVA and Sidak's multiple comparison test (H); *p < 0.05 versus HBEC3‐KT cells; **p < 0.01 compared to the adjacent tissues; #*p* < 0.05 compared to the low circ_515 expression group or the control group.

DE circRNAs between NSCLC and normal lung tissues were screened using the GSE158695 dataset (Figure 1A). A total of 114 DE circRNAs were obtained, among which 37 were increased whereas 77 were decreased in NSCLC (Figure 1B). The DE genes were ranked by log FC and *adj. p* values to screen the circRNAs with high correlation with NSCLC development. It was observed that hsa_circRNA_000585 ranked in the top three both in terms of the log FC value and the *adj. p‐value* (Figure 1C). In the circBase system (http://circbase.org/), the circbase ID of hsa_circRNA_000585 is hsa_circ_0000515 (circ_515). Based on the ID, we obtained the sequence information of circ_515 in the circBank system. hsa_circRNA_000585 sequence is 229 bp in length and is generated by back‐splicing of the mRNA of the host gene RPPH1 (Figure 1D).

Next, the abundance of circ_515 in the tissues was examined using RT‐qPCR. Of note, circ_515 expression was enhanced in the NSCLC tissues versus the adjacent tissues (Figure 1E). Based on the mean value of circ_515 expression (2.4), the patients were assigned to the high‐circ_515 group (*n* = 25) and low‐circ_515 expression groups (*n* = 25). The 5‐year survival of patients was recorded. High expression of circ_515 was associated with worse survival of patients (Figure 1F). Moreover, elevation of circ_515 was correlated with advanced tumor node metastasis stage, poor tumor differentiation, and increased lymph node metastasis (Table 3). Likewise, increased circ_515 expression was observed in the NSCLC cell lines compared to the HBEC3‐KT cells (Figure 1G). The SK‐MES‐1 cells with the highest circ_515 expression and the A549 cells with the lowest expression were selected for subsequent experiments.

**TABLE 3:** Clinicopathological characteristics of patients with NSCLC.

		Cases (*n* = 50)	circ_515 expression		*p*‐value
Clinicopathological parameters			Low (*n* = 25)	High (*n* = 25)	
Gender	Male	29	13	16	0.5674
	Female	21	12	9	
Age	≥ 60	30	14	16	0.7733
	< 60	20	11	9	
Tumor size	≥ 4	22	9	13	0.3931
	< 4	28	16	12	
TNM	I–II	26	18	8	*0.0101
	III/IV	24	7	17	
Lymph metastasis	Positive	22	7	15	*0.0450
	Negative	28	18	10	
Smoke	No	11	7	4	0.4962
	Yes	39	18	21	
Differentiation	Well/moderate	29	20	9	**0.0037
	Poor	21	5	16	

*Note*: Clinical information was analyzed by Fisher’s exact test.

Abbreviations: NSCLC, non‐small‐cell lung cancer; TNM, tumor node metastasis.

*p < 0.05 and **p < 0.01 represents statistical difference.

Because of their lack of open ends, circRNAs are resistant to RNase R treatment and are more stable than their linear transcripts.^8^ To examine the stability of circ_515, the SK‐MES‐1, and A549 cells were treated with RNase R, after which the mRNA of host gene RPPH1 was degraded, but the circ_515 was not significantly affected (Figure 1H).

### 3.2 Circ_515 enhances the viability of NSCLC cells

**FIGURE 2:**
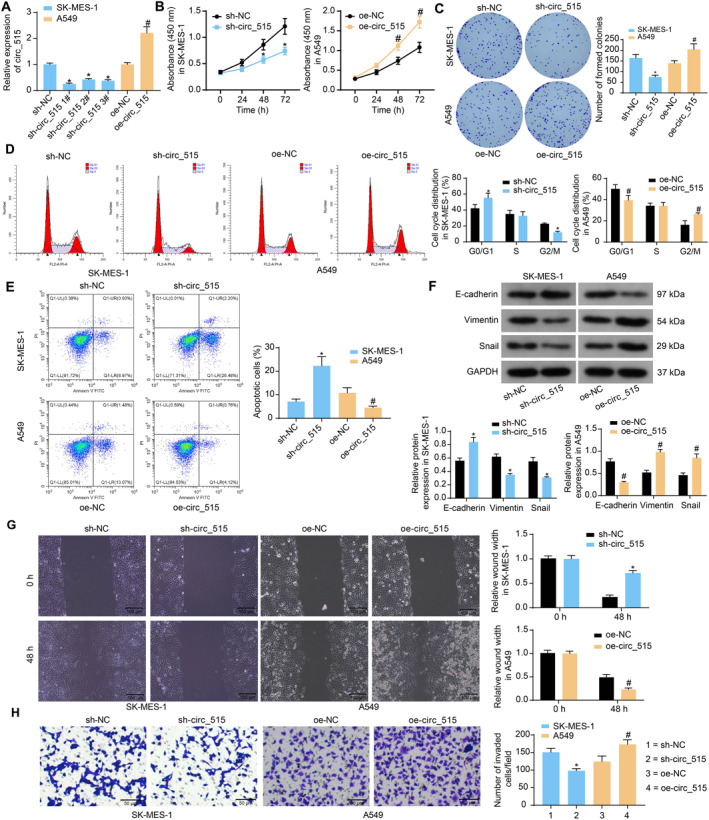
Overexpression of circ_515 enhances whereas downregulation of circ_515 suppresses malignant behaviors of NSCLC cells. (A), circ_515 expression in SK‐MES‐1 and A549 cells after sh‐ circ_515 or oe‐circ_515 transfection determined by RT‐qPCR; (B), proliferation ability of SK‐MES‐1 and A549 cells determined by the CCK‐8 assay; (C), colony formation ability of SK‐MES‐1 and A549 cells detected by colony formation assay; (D and E), cell cycle distribution (D) and apoptosis (E) of cells examined by flow cytometry. (F), protein levels of the epithelial‐mesenchymal transition (EMT)‐related proteins in SK‐MES‐1 and A549 cells examined by western blot analysis; (G), migration of SK‐MES‐1 and A549 cells determined by wound‐healing assay; (H), invasion of SK‐MES‐1 and A549 cells determined by transwell assay. Data were collected from three independent experiments and expressed as mean ± SD. Differences were analyzed by one‐way ANOVA and Tukey’s multiple comparison tests (A, C, E, and H) or two‐way ANOVA and Sidak’s multiple comparison tests (B, D, F, and G); *p < 0.05 compared to sh‐NC, #*p* < 0.05 compared to oe‐NC.

To validate the exact role of circ_515 in NSCLC, shRNAs of circ_515 (sh‐circ_515 1, 2, 3#) were transfected into SK‐MES‐1 cells, and oe‐circ_515 was transfected into A549 cells. The efficacy was confirmed 48 h later using RT‐qPCR (Figure 2A). The sh‐circ_515 1# was used for the following experiments due to its outstanding efficacy.

After circ_515 expression interference, the CCK‐8 method was conducted. Downregulation of circ_515 suppresses the proliferation ability of SK‐MES‐1 cells. In contrast, the upregulation of circ_515 in A549 cells enhanced cell proliferation (Figure 2B). The colony formation of SK‐MES‐1 cells was found to be inhibited but that of the A549 cells was encouraged (Figure 2C). The flow cytometry indicated that silencing of circ_515 induced the cell cycle arrest at G0/G1 phases of SK‐MES‐1 cells. Upregulation of circ_515 resulted in A549 cell arrest at G2/M phases (Figure 2D). In addition, the apoptosis of SK‐MES‐1 was increased whereas that of the A549 cells was reduced (Figure 2E). Moreover, the EMT in cells was analyzed. Silencing of circ_515 elevated the level of E‐cadherin, whereas suppressed the levels of vimentin and snail in SK‐MES‐1 cells, and the circ_515 overexpression resulted in inverse trends in A549 cells (Figure 2F). The 48‐h width of the scratch on SK‐MES‐1 cells increased, whereas that on A549 cells decreased (Figure 2G), indicating that circ_515 enhances the migration of NSCLC cells. Meanwhile, the transwell assay indicated that the circ_515 downregulation reduced, whereas circ_515 overexpression enhanced the invasion of the NSCLC cells (Figure 2H).

### 3.3 Circ_515 acts as a sponge for miR‐296‐5p

**FIGURE 3:**
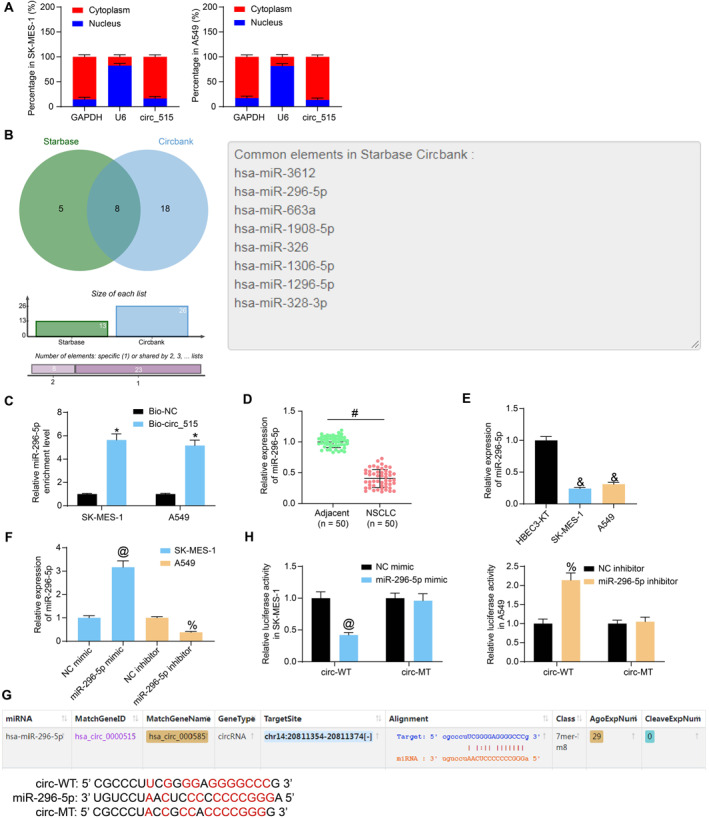
Circ_515 serves as a sponge for miR‐296‐5p. (A), sub‐cellular localization of circ_515 in SK‐MES‐1 and A549 cells examined by nuclear‐cytoplasmic RNA separation assay; (B), candidate miRNAs possibly binding to circ_515 predicted using circBank and Starbase; (C), miRNA sponged by the bio‐circ probe examined by RNA pull‐down assay; (D), expression of miR‐296‐5p in clinically collected NSCLC tissues examined by RT‐qPCR; (E), expression of miR‐296‐5p in NSCLC cells examined by RT‐qPCR; (F), transfection efficacy of miR‐296‐5p mimic/inhibitor in NSCLC cells examined by RT‐qPCR; (G), the putative binding sequence between circ_515 and miR‐296‐5p and the MT sequence used for luciferase assay; (H), activity of the circ‐WT/circ‐MT luciferase reporter vectors in SK‐MES‐1 and A549 cells after miR‐296‐5p mimic or inhibitor transfection examined by the luciferase reporter gene assay. Data were collected from three independent experiments and expressed as mean ± SD. Differences were analyzed by paired *t‐test* (D), one‐way ANOVA (Dunnett’s multiple comparisons test for E and Tukey’s multiple comparisons test for [F]), or two‐way ANOVA and Sidak’s multiple comparison tests (A, C, and H); *p < 0.05 compared to bio‐NC, #*p* < 0.05 fibroblast growth factor receptor 1 compared to adjacent tissues; & *p* < 0.05 compared to HBEC3‐KT, @*p* < 0.05 compared to NC mimic, %*p* < 0.05 versus NC inhibitor.

To expound the molecular mechanism involved, the sub‐cellular localization of circ_515 in NSCLC was first examined. Circ_515 was principally localized in the cytoplasm of NSCLC cells (Figure 3A), indicating that it may sponge specific miRNAs. We then explored the candidate target miRNAs of circ_515 via the circBank and Starbase (http://starbase.sysu.edu.cn/) systems. Eight candidate mRNAs were obtained, including hsa‐miR‐3612, hsa‐miR‐296‐5p, hsa‐miR‐663a, hsa‐miR‐1908‐5p, hsa‐miR‐326, hsa‐miR‐1306‐5p, hsa‐miR‐1296‐5p, and hsa‐miR‐328‐3p (Figure 3B). Among them, miR‐296‐5p was downregulated in NSCLC tissues and cells, and it repressed the malignant behavior of cancer cells by suppressing the downstream targets.^17^ RNA pull‐down assay was performed, and the enrichment of candidate miRNAs was examined by the probe bio‐circ with biotin‐coupled probe circ_515 in NSCLC cells using RT‐qPCR (Figure 3C, Figure S1A). miR‐296‐5p was the most significantly enriched miRNA, so we chose miR‐296‐5p as our study subject. Thereafter, the RT‐qPCR identified decreased miR‐296‐5p expression in the tumor tissues (Figure 3D). Alike, decreased miR‐296‐5p expression was observed in the NSCLC cells (Figure 3E).

Afterward, the miR‐296‐5p mimic and NC mimic were delivered into SK‐MES‐1 cells, whereas the miR‐296‐5p inhibitor and NC inhibitor were delivered into A549 cells (Figure 3F). The binding sequence between circ_515 and miR‐296‐5p was downloaded from Starbase, and the MT sequence was constructed (Figure 3G). miR‐296‐5p mimic notably suppressed the activity of circ‐WT luciferase vector in SK‐MES‐1 cells, whereas miR‐296‐5p inhibitor elevated the activity of circ‐WT luciferase vector in A549 cells (Figure 3H).

### 3.4 miR‐296‐5p mimic reduces malignant properties of NSCLC cells

**FIGURE 4:**
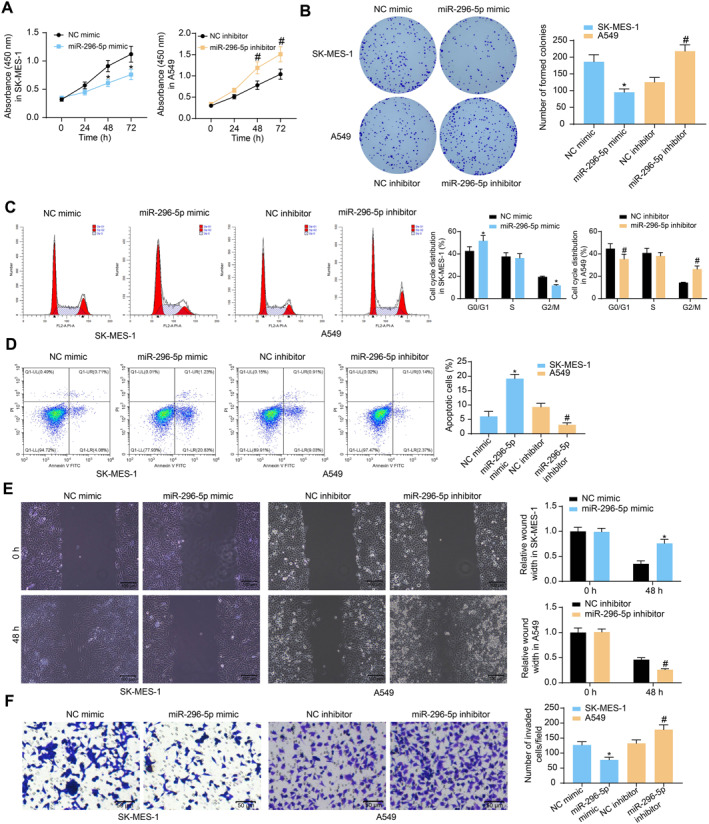
miR‐296‐5p mimic reduces whereas miR‐296‐5p inhibitor enhances malignant properties of NSCLC cells. (A), proliferation of SK‐MES‐1 and A549 cells after miR‐296‐5p mimic or miR‐296‐5p inhibitor transfection examined by CCK‐8 method; (B), colony formation ability of SK‐MES‐1 and A549 cells determined by the colony formation assay; (C and D), cell cycle distribution (C) and apoptosis (D) of SK‐MES‐1 and A549 cells detected by flow cytometry; (E), migration ability of SK‐MES‐1 and A549 cells examined by the wound‐healing assay; (F), invasion ability of SK‐MES‐1 and A549 cells detected by the transwell assay. Data were collected from three independent experiments and expressed as mean ± SD. Differences were analyzed by one‐way ANOVA and Tukey's multiple comparisons tests (B, D, and F) or two‐way ANOVA and Sidak's multiple comparison tests (A, C, and E); *p < 0.05 compared to NC mimic, #*p* < 0.05 compared to NC inhibitor.

The SK‐MES‐1 cells transfected with miR‐296‐5p mimic and A549 cells transfected with miR‐296‐5p inhibitor were used for behavioral analysis. The proliferation of cells was repressed by miR‐296‐5p overexpression but enhanced by miR‐296‐5p inhibition (Figure 4A). Similarly, the colony formation of SK‐MES‐1 cells was suppressed but that of A549 cells was promoted (Figure 4B). According to the flow cytometry, miR‐296‐5p mimic induced cell cycle arrest at G0/G1 phases, but the miR‐296‐5p inhibition promoted more cells to enter the G2/M phases (Figure 4C). Moreover, miR‐296‐5p mimic enhanced whereas the miR‐296‐5p inhibitor reduced the number of apoptotic NSCLC cells (Figure 4D). Lastly, the NSCLC cell migration and invasion potentials were suppressed in SK‐MES‐1 cells but enhanced in A549 cells (Figure 4E,F).

### 3.5 miR‐296‐5p targets RNF44

**FIGURE 5:**
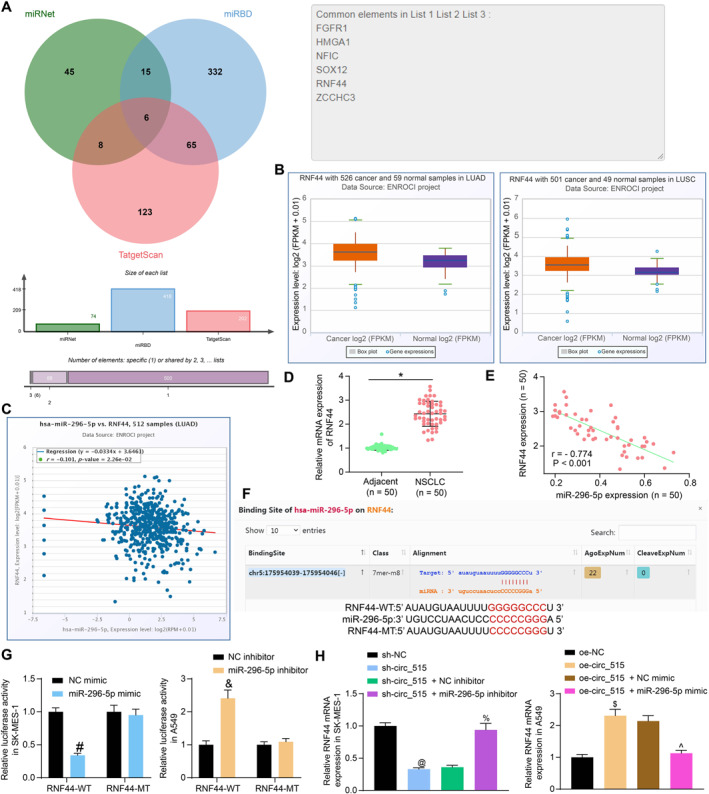
miR‐296‐5p targets RNF44. (A), candidate target genes of hsa‐miR‐296‐5p predicted using several bioinformatic systems; (B), expression of RNF44 in NSCLC using the Pan‐cancer platform on the Starbase system; (C), a negative correlation between RNF44 and miR‐296‐5p in LUAD predicted in the Starbase Pan‐cancer system; (D), RNF44 expression in collected NSCLC tissues detected by RT‐qPCR; (E), a negative correlation between RNF44 and miR‐296‐5p in NSCLC tissues evaluated by Pearson's correlation analysis (*r* = ‐ 0.774, *p* < 0.001); (F), putative binding sequence between RNF44 and miR‐296‐5p and the MT sequence used for luciferase assay; (G), binding relationship between RNF44 and miR‐296‐5p validated using the luciferase reporter gene assay; (H), RNF44 expression in SK‐MES‐1 and A549 cells with different transfections analyzed by RT‐qPCR. Data were collected from three independent experiments and expressed as mean ± SD. Differences were analyzed by paired *t‐test* (D), one‐way ANOVA and Tukey’s multiple comparisons tests (H), or two‐way ANOVA and Sidak’s multiple comparison tests (G); *p < 0.05 compared to adjacent tissue, #*p* < 0.05 compared to NC mimic, &*p* < 0.05 compared to NC‐inhibitor, @*p* < 0.05 compared to sh‐NC, %*p* < 0.05 compared to sh‐circ_515 + NC inhibitor, $*p* < 0.05 compared to oe‐NC, ^*p* < 0.05 compared to oe‐circ_515 + NC mimic.

The targets of miR‐296‐5p were predicted using several bioinformatic tools including miRBD (http://mirdb.org/mirdb/index.html), miRNet (https://www.mirnet.ca/miRNet/home.xhtml), and TargetScan8.0 (https://www.targetscan.org/vert_80/). Six genes including fibroblast growth factor receptor 1 (FGFR1), HMGA1, nuclear factor 1 C‐type (NFIC), SOX12, RNF44, and ZCCHC3 were suggested to be intersected among the three sets of genes (Figure 5A). First, the expression of the six intersecting genes in LUSC and LUAD was examined, and the results showed that only FGFR1, HMGA1, NFIC, SOX12, and RNF44 were DE in both LUAD and LUSC. Since miR‐296‐5p was poorly expressed in NSCLC, we excluded FGFR1 and NFIC, which have reduced expression in LUSC and LUAD. Among the remaining SOX12, RNF44, and HMGA1, we further analyzed the relation between miR‐269‐5p expression and the expression of the three targets in NSCLC. Only RNF44 had a negative correlation in LUAD and LUSC, and SOX12 or HMGA1 were positively correlated or not correlated with miR‐296‐5p. Therefore, we chose RNF44 as the research object (Figure 5B,C, Figure S1B,C). The subsequent RT‐qPCR validated that RNF44 was overexpressed in the NSCLC tissues (Figure 5D), which showed a converse relation with miR‐296‐5p (Figure 5E).

The putative binding site between RNF44 and miR‐296‐5p was downloaded from TargetScan and the MT sequence was designed (Figure 5F). The transfection of miR‐296‐5p mimic lowered luciferase activity of the RNF44‐WT luciferase vector in SK‐MES‐1 cells, while miR‐296‐5p inhibitor enhanced the luciferase activity of the RNF44‐WT luciferase vector in A549 cells (Figure 5G). Thereafter, the SK‐MES‐1 cells transfected with sh‐circ_515 were further treated with miR‐296‐5p inhibitor, whereas A549 cells transfected with oe‐circ_515 were additionally transfected with miR‐296‐5p mimic for rescue experiments. The RT‐qPCR presented that sh‐ circ_515 reduced RNF44 expression, but these trends were reversed by the miR‐296‐5p inhibitor. Correspondingly, the RNF44 expression was elevated by oe‐circ_515, and the miR‐296‐5p mimic led to converse changes (Figure 5H).

### 3.6 RNF44 is responsible for the oncogenic events mediated by circ_515

**FIGURE 6:**
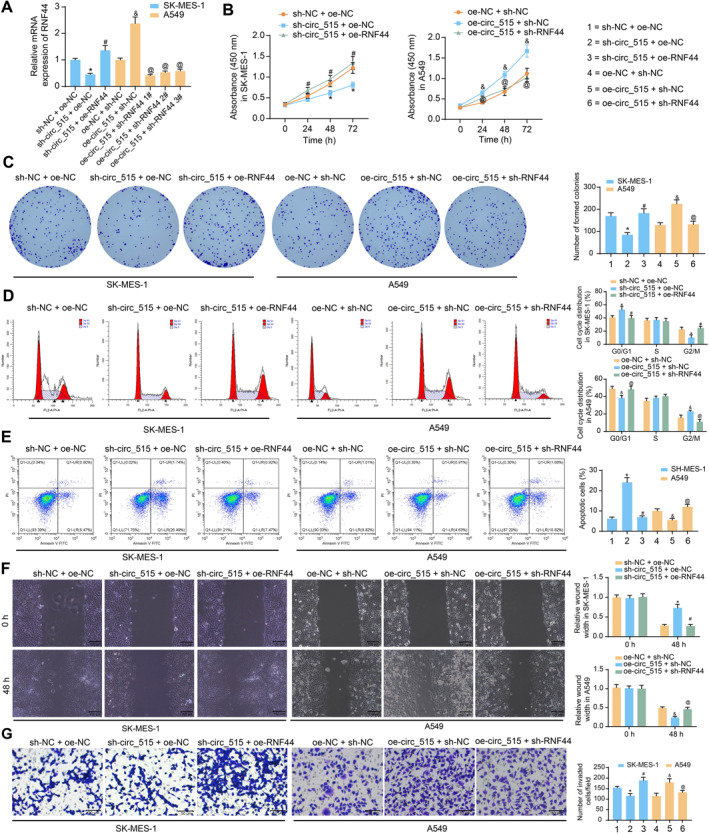
RNF44 is responsible for the oncogenic events mediated by circ_515. (A), expression of RNF44 mRNA in SK‐MES‐1 and A549 with further oe‐RNF44 or sh‐RNF44 transfections examined by RT‐qPCR; (B), proliferation of SK‐MES‐1 and A549 cells after further oe‐RNF44 or sh‐RNF44 transfection examined by CCK‐8 method; (C), colony formation ability of SK‐MES‐1 and A549 cells detected by the colony formation assay; (D and E), cell cycle distribution (D) and apoptosis (E) of SK‐MES‐1 and A549 cells examined by flow cytometry; (F), migration ability of SK‐MES‐1 and A549 cells examined by the wound‐healing assay; (G), invasion ability of SK‐MES‐1 and A549 cells detected by the transwell assay. Data were collected from three independent experiments and expressed as mean ± SD. Differences were analyzed by one‐way ANOVA and Tukey’s multiple comparison tests (A, C, E, and G) or two‐way ANOVA and Sidak's multiple comparison tests (B, D, and F); *p < 0.05 compared to sh‐NC + oe‐NC, #*p* < 0.05 compared to sh‐circ 515 + oe‐NC, &*p* < 0.05 compared to oe‐NC + sh‐NC; @*p* < 0.05 compared to oe‐circ 515 + sh‐NC.

To further identify the implication of RNF44 in circ_515‐mediated events, we transfected SK‐MES‐1 cells with sh‐NC + oe‐NC, sh‐circ_515 + oe‐NC, and sh‐circ_515 + oe‐RNF44 or transfected A549 cells with sh‐NC + oe‐NC, oe‐circ_515 + sh‐NC, and oe‐circ_515 + sh‐RNF44 1#, 2#, 3#. The successful transfections were verified using RT‐qPCR (Figure 6A), and the sh‐RNF44 1# was used in the following assays. The proliferation and colony formation of SK‐MES‐1 cells repressed by sh‐circ_515 were recovered by oe‐RNF44, and those of A549 cells were repressed after sh‐RNF44 (Figures 6B,C). Moreover, the flow cytometry indicated that oe‐RNF44 significantly triggered the cell cycle progression and hampered apoptosis of SK‐MES‐1, whereas sh‐RNF44 induced cell cycle arrest at G0/G1 phases and accordingly increased the apoptosis of A549 cells (Figures 6D,E). Overexpression of RNF44 also restored the migratory and invasive potential of SK‐MES‐1 cells, whereas downregulation of RNF44 reduced the migration and invasiveness of A549 cells that were induced by sh‐circ_515 (Figures 6F,G).

### 3.7 Silencing of circ_515 slows growth of xenografts in mice

**FIGURE 7:**
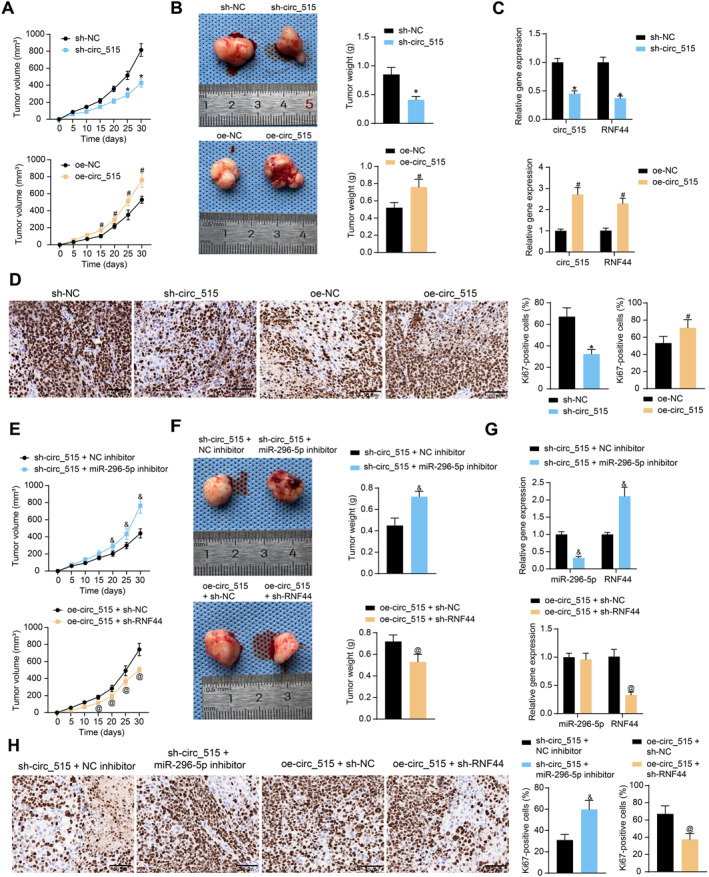
Knockdown of circ_515 reduces growth of xenograft tumors in vivo. (A), volume change of the xenograft tumors formed by SK‐MES‐1 cells knocking down circ_515 and A549 cells overexpressing circ_515; (B), weight of the xenograft tumor on the 30th day; (C), expression of circ_515 and RNF44 mRNA in the xenograft tumors examined by RT‐qPCR; (D), protein level of KI‐67 in the xenograft tumors examined by IHC; (E), volume change of the xenograft tumors formed by SK‐MES‐1 cells with sh‐circ_515 + miR‐296‐5p inhibitor and A549 cells with oe‐circ_515 + sh‐RNF44; (F), weight of the xenograft tumor on the 30th day; (G), expression of miR‐296‐5p and RNF44 mRNA in the xenograft tumors examined by RT‐qPCR; (H), protein level of KI‐67 in the xenograft tumors examined by IHC. In each group, *n* = 5; Data were expressed as the mean ± SD. Differences were analyzed by unpaired *t‐test* (B, D, F, H) or two‐way ANOVA and Sidak’s multiple comparison tests (A, C, E, G); *p < 0.05 compared to sh‐NC, #*p* < 0.05 compared to oe‐NC, &*p* < 0.05 compared to sh‐circ_515 + NC inhibitor; @*p* < 0.05 compared to oe‐circ_515 + sh‐NC.

We selected SK‐MES‐1 cells infected with sh‐NC or sh‐circ_515 group and A549 cells infected with oe‐NC or oe‐circ_515 to verify the effect of circ_515 on tumor growth in vivo. Sh‐circ_515 reduced the growth rate of SK‐MES‐1 cells, while oe‐circ_515 accelerated the growth of A549 cells in vivo (Figure 7A). On the 30th day, the tumors were dissected. Sh‐circ_515 was found to reduce the weight of xenograft tumors, whereas tumors formed by A549 cells overexpressing A549 cells were heavier (Figure 7B). In the collected tissues, importantly, we confirmed that sh‐circ_515 downregulated circ_515 and the mRNA expression of RNF44 (Figure 7C), as well as the expression of KI‐67 in xenografts (Figure 7D). By contrast, oe‐circ_515 increased the expression of circ_515, RNF44, and KI‐67 (Figure 7C,D).

Next, SK‐MES‐1 cells treated with sh‐circ_515 + NC inhibitor or sh‐circ_515 + miR‐296‐5p inhibitor and A549 cells treated with oe‐circ_515 + sh‐NC, oe‐circ_515 + sh‐RNF44 were injected into mice for functional rescue experiments. The tumor growth was accelerated and tumor weight was increased following sh‐circ_515 + miR‐296‐5p inhibitor treatment, whereas tumor growth was inhibited and tumor weight was reduced in the oe‐circ_515 + sh‐RNF44 group compared to the oe‐circ_515 + sh‐NC group (Figure 7E,F). Decreased miR‐296‐5p expression and increased RNF44 expression was observed in tumor tissues with sh‐circ_515 + miR‐296‐5p inhibitor. In contrast, miR‐296‐5p expression did not show significant changes and RNF44 expression significantly decreased in the tumor tissues with oe‐circ_515 + sh‐RNF44 (Figure 7G). IHC showed an increased rate of Ki67‐positive cells in tumor tissues with sh‐circ_515 + miR‐296‐5p inhibitor, whereas the rate of Ki67 positivity decreased in the oe‐circ_515 + sh‐RNF44 group (Figure 7H).

## 4 DISCUSSION

Identifying alterations in key driver genes and exploring novel molecular mechanisms involved in tumorigenesis are important issues to offer more opportunities and increase the survival of patients.^19^ In this study, we reported an unreported circ_515/miR‐296‐5p/RNF44 axis which may be closely linked to the pathogenesis of NSCLC.

CircRNAs are formed by back‐splicing during which a downstream 5′ splice site is joined to an upstream 3′ splice site across a single or multiple exons, and the absence of free ends leads to a higher stability of circRNAs than linear RNAs.^7^ The circRNAs exert varying biological functions and are versatile molecular regulators as well as novel diagnostic and prognostic markers in human cancers including NSCLC.^20^ For instance, upregulation of circ_000984 promoted proliferation and metastasis of NSCLC cells via the Wnt/*β*‐catenin signaling.^21^ By contrast, hsa_circ_0008305 was found to reduce transforming growth factor *β*‐mediated EMT of NSCLC cells.^22^ In the GSE158695 dataset from the GEO database, circ_515 was identified as a substantially increased circRNA in NSCLC. In the work by Yi et al., the authors detected upregulated circ_515 in cholangiocarcinoma patients, though the circ_515 was found to have a nonsignificant correlation with the clinicopathological characteristics of patients.^13^ Here, we revealed significantly upregulated circ_515 expression in NSCLC tumors and cells and confirmed its correlation with lymph metastasis, TNM stage, and poor tumor differentiation of patients. Moreover, we performed functional blockade assays and unraveled that the circ_515 knockdown suppressed the proliferation, colony formation, apoptosis resistance, cell cycle entry, mobility, and tumorigenicity of NSCLC cells.

The cytoplasm‐localization of circ_515 indicated that it might serve as a ceRNA for other transcripts. In this work, we obtained by bioinformatic analysis and the RNA pull‐down assay that circ_515 interacted with miR‐296‐5p, which was poorly expressed in NSCLC samples. miR‐296‐5p has been recognized as a sponge for several circRNAs such as circNSUN2 ^23^ and circPLK1, ^24^ and its restoration suppressed the growth and aggressiveness of different kinds of cancer cells. miR‐296‐5p has been identified to be a diagnostic biomarker for thyroid cancer.^25^ In the same vein, reduced miR‐296‐5p was detected in NSCLC tissues and its restoration blocked the malignant phenotype of cancer cells.^26^ In concert with these previous reports, we identified miR‐296‐5p downregulation both in NSCLC tissues and cell lines, and the miR‐296‐5p mimic suppressed the malignant properties of cancer cells. The miR‐296 precursor generally gives rise to mature miR‐296 which is called miRNA‐296‐5p if derived from the 5′ arm and miRNA‐296‐3p if derived from the 3′ arm.^27^ More recently, miR‐296‐3p has been linked to response to high iodine and radiosensitivity in papillary thyroid cancer and pancreatic cancer, respectively.^28^, ^29^ Likewise, miR‐296‐5p upregulation sensitized nasopharyngeal carcinoma cells to cisplatin by suppressing cell proliferation and colony formation and supporting apoptosis.^30^ All these findings suggested an association between miR‐296‐5p and response to treatment in cancers, which might be the focus of our next study.

Among the targets of miR‐296‐5p, highly expressed SOX12, RNF44, and HMGA1 piqued our attention. Even though SOX12 was not related to miR‐296‐5p and HMGA1 was positively correlated with miR‐296‐5p, their interactions were reported in other cancers.^31^, ^32^, ^33^ By performing integrated bioinformatic analyses and luciferase assay again, we identified RNF44 as a target of miR‐296‐5p in NSCLC. RNF44 is a member of the E3 ubiquitin ligases, which induce the ubiquitination of a myriad of protein substrates for targeted degradation and are increasingly considered anticancer targets.^34^, ^35^ Although there is no document concerning the correlation between RNF44 and NSCLC, other members of the RNF families such as RNF19A ^36^ and RNF38 ^37^ have been clearly defined to be upregulated in NSCLC samples and lead to malignant development of disease. In this work, we identified increased RNF44 expression in NSCLC tissues and cells. Most of all, the artificial RNF44 upregulation rescued the malignant properties of NSCLC cells repressed by circ_515 knockdown, indicating that RNF44 is at least partly involved in the oncogenic properties mediated by circ_515.

**FIGURE 8:**
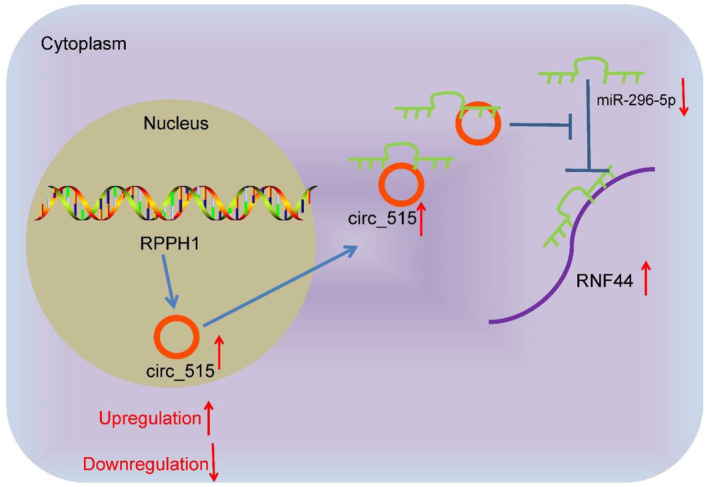
A diagram for molecular mechanism. In NSCLC cells, circ_515 sequesters miR‐296‐5p and blocks its suppressive function on RNF44, leading to cancer progression.

In conclusion, circ_515 may function as a prognostic marker for NSCLC whose upregulation enhanced the growth and invasiveness of cancer cells by interacting with miR‐296‐5p and activating RNF44 (Figure 8). These findings may provide new insights into the treatment of NSCLC, as circ_515 and RNF44 may serve as therapeutic targets for NSCLC treatment. Nevertheless, the targets of RNF44, as an E3 ligase, in the NSCLC development remain vague in the present work. The target molecules or signaling of the circ_515/miR‐296‐5p/RNF44 signaling will be examined in our following studies.

## AUTHOR CONTRIBUTIONS

**Lixin Sun:** Conceptualization, writing—original draft, formal analysis, project administration. **Bei Lu:** Validation, writing—review and editing, investigation, methodology. **Chongyuan Li:** Resources, writing—original draft, data curation, software. **Guangquan Xu:** Writing—review and editing, supervision, formal analysis, visualization.

## CONFLICT OF INTEREST STATEMENT

The authors declare no conflicts of interest.

## ETHICS STATEMENT

The study was approved by the Ethical Committee of the Fourth Affiliated Hospital of Harbin Medical University. An informed consent form was obtained from each respondent. Animal procedures were approved by the Animal Committee of the Fourth Affiliated Hospital of Harbin Medical University and performed strictly adhering to the Guide for the Care and Use of Laboratory Animals (NIH, Bethesda).

## Supporting information

Supporting Information S1

Figure S1

## Data Availability

Data will be made available on request.
