# Supplementary material for: Hsa_circ_0000515 sequesters microRNA‐296‐5p and elevates RNF44 expression to encourage the NSCLC progression
Source: J Cell Commun Signal. 2025 Feb 24;19(1):e70005. doi: 10.1002/ccs3.70005 (PMC11850089; doi:10.1002/ccs3.70005)
Supplement: Supplementary file 1 — Supporting Information S1 [file CCS3-19-e70005-s001.docx]

**Supporting Information**

**Cell transfection**

Lentiviral vectors harboring circ_515 and RNF44 (oe-circ_515 and oe-RNF44), shRNAs of circ_515 and RNF44 (sh- circ_515 1, 2, 3# and sh-RNF44 1, 2, 3#), and the negative control (oe-NC and sh-NC) were procured from VectorBuilder (Guangzhou, Guangdong, China). The miR-296-5p mimic (AGGGCCCCCCCUCAAUCCUGU), miR-296-5p inhibitor (CAGGATTGAGGGGGGGCC), and the controls were acquired from GenePharma Co., Ltd. (Shanghai, China). Cells were infected with lentiviral vectors and transfected according to the instruction manuals of a Lipofectamine 2000 kit (Thermo Fisher Scientific Inc., Waltham, MA, USA).

CCK-8

The NSCLC cells seeded in 96-well plates at 3,000 cells per well were supplemented with 10 μL CCK-8 solution (Beyotime, Shanghai, China) at 0, 24, 48, and 72 h, respectively, and cultured for 120 min. The absorbance at 450 nm was read using a microplate reader (Bio-Tek Company, Winooski, VT, USA).

Colony formation assay

NSCLC cells were cultured in 6-well plates at 1,000 cells per well and cultured under a standard condition for 2 weeks. After that, the cells were fixed in formaldehyde (Solarbio, Beijing, China) for 15 min and stained with 0.1% crystal violet (Beyotime) for 1 h. The number of cell colonies was observed and counted under a microscope (Olympus Optical Co., Ltd, Tokyo, Japan).

Flow cytometry

For cell cycle detection, the transfected NSCLC cells (2 × 10^5^ cells) were detached in trypsin and fixed in 4% formaldehyde overnight. Next, the cells were centrifuged at 300 g for 5 min, treated with RNase A (0.1 mg/mL) and PI (0.05 mg/mL, Sigma-Aldrich) at 20°C for 20 min, and loaded onto a FACS cytometer (BD Biosciences, NJ, USA). Modfit 5.0 software was used for analysis. In short, the FCS file obtained from FACS flow cytometry analysis was imported into Modfit 5.0 software, and the parameters were set and adjusted for the simulation and integration of cell cycle distribution maps. Finally, the proportion of cells in each phase was analyzed.

For apoptosis detection, the cells were cultured in 6-well plates at 6 × 10^5^ cells per well. Next, the cells were warm-incubated for 2 d and stained using an Annexin V/PI staining kit (BD Biosciences). The apoptotic cells (defined as Annexin V^+^PI^-^) were examined using the flow cytometer.

Migration assay

Transfected cells were cultured in 6-well plates. When the cell confluence reached 80%-90%, a sterile 10-μL pipette tip was applied to produce scratches on the cell monolayer. Next, the cell debris was rinsed by PBS, and the remaining cells were cultured in an FBS-free medium for 48 h. The images of scratch at 0 h and 48 h were captured, and the scratch width was analyzed using Image J. The loaded images were subjected to contrast enhancement (Saturated pixels = 0.3%), followed by smoothing and edge finding operation on the image. After thresholding, a wand tool was used to select the wound, and the Analyze-Measure option was chosen to get the wound area. Finally, the wound area was divided by the height to get the wound width.

Transwell assay

A Transwell assay using 24-well Matrigel-coated chambers (Corning Glass Works, Corning, NY, USA). In short, 5 × 10^4^ NSCLC cells were resuspended in 300 µL FBS-free medium and loaded in apical chambers. The basolateral chambers were filled with 700 μL 10% FBS-supplemented medium. After 24 h of culture at 37°C, invaded cells were fixed in formaldehyde and stained with 0.5% crystal violet for 60 min. The number of invaded cells was counted under an inverted microscope (Olympus).

Dual-luciferase assays

The putative binding sequence (wild-type, WT) between circ_515 and miR-296-5p was obtained from circBank (www.circBank.cn), and the binding sequence between miR-296-5p and RNF44 3’UTR was obtained from TargetScan (http://www.targetscan.org/vert_72/). The mutant-type (MT) sequences were designed. The sequences were cloned into pGL3 luciferase vectors (Promega, Fitchburg, WI, USA) to design circ-WT/circ-MT and RNF44-WT/RNF44-MT luciferase reporter vectors. Well-constructed vectors were co-transfected with NC mimic or miR-296-5p mimic into SK-MES-1 cells, or co-transfected with miR-296-5p inhibitor or NC inhibitor into A549 cells. After 48 h, a dual-luciferase reporter assay system (Promega) was used to assess luciferase activity.

Immunohistochemistry (IHC)

The collected tumor tissues were fixed in 10% formalin, paraffin-embedded, and cut into 5-μm sections. The sections were co-incubated with anti-KI-67 (1:200, ab16667, Abcam) at 4℃ overnight. The nuclei were counter-stained with hematoxylin (Sigma-Aldrich). The staining was observed and photographed under an Axiophot microscope (Zeiss, Oberkochen, Germany). The positive staining rate was calculated using Image J. Briefly, after changing the image to RGB stack format, the positive signal was selected under the threshold option to count the number of positive cells. The positive cell rate (%) was defined as the number of positive cells to the total number of cells.
