# Supplementary material for: Hsa_circ_0000515 sequesters microRNA‐296‐5p and elevates RNF44 expression to encourage the NSCLC progression
Source: J Cell Commun Signal. 2025 Feb 24;19(1):e70005. doi: 10.1002/ccs3.70005 (PMC11850089; doi:10.1002/ccs3.70005)
Supplement: Supplementary file 2 — Figure S1 [file CCS3-19-e70005-s002.docx]

**
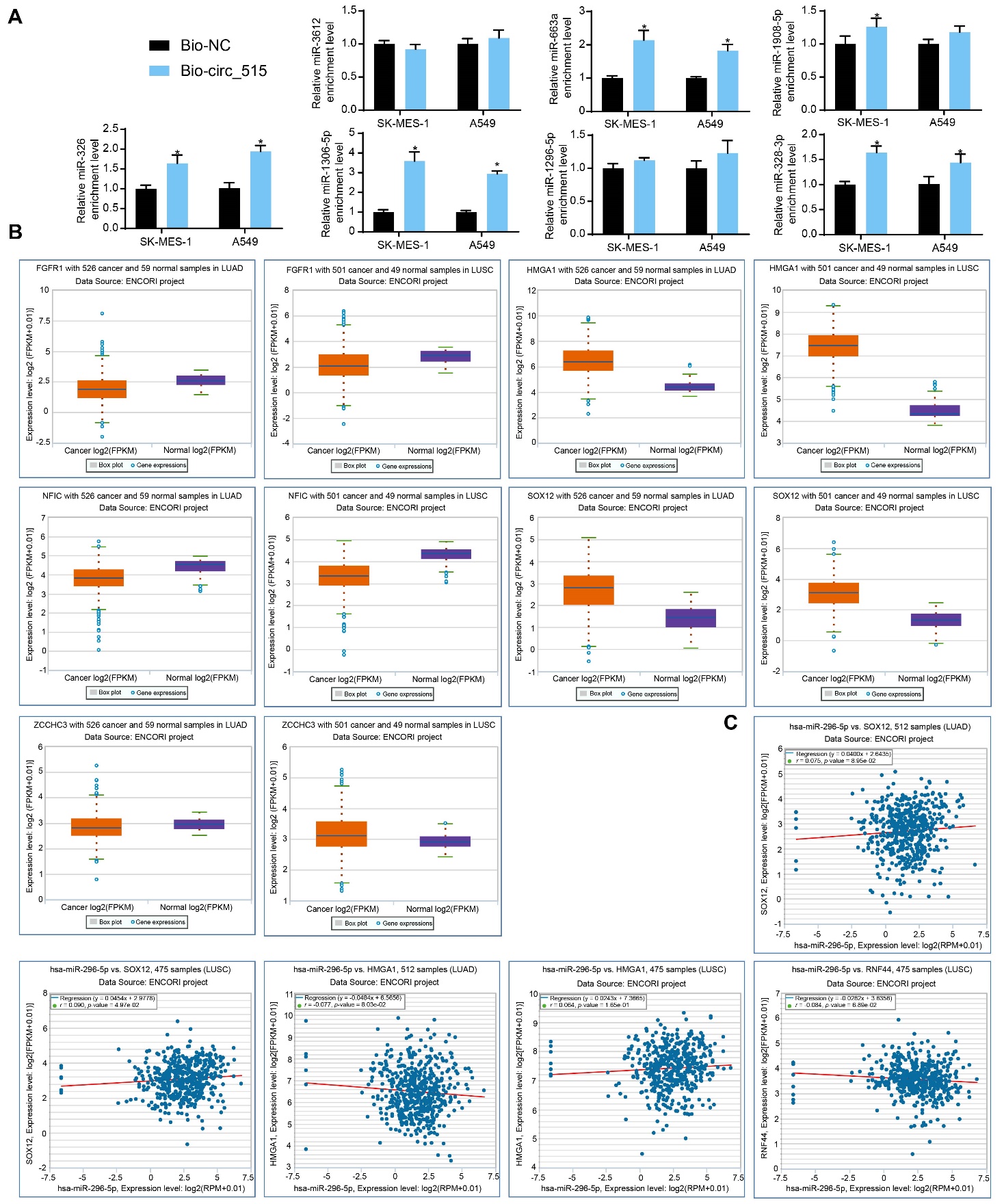
**

**Supplementary Figure 1** Screening for downstream miRNAs of circ_515 and target mRNAs of miR-296-5p. A, Enrichment of miR-3612, miR-663a, miR-1908-5p, miR-326, miR-1306-5p, miR-1296-5p, and miR-328-3p in RNA pull-down experiments to analyze biotinylated Circ_515 pull down miRNAs; B, Expression of FGFR1, HMGA1, NFIC, SOX12, and ZCCHC3 in LUSC and LUAD analyzed by Starbase database; C, Correlation of miR-296-5p with SOX12, RNF44, and HMGA1 expression in LUSC and LUAD analyzed by Starbase database. Differences were analyzed by two-way ANOVA and Sidak's multiple comparison tests (A); **p* < 0.05 compared to Bio-NC.
